# Supplementary material for: Borrelia burgdorferi loses essential genetic elements and cell proliferative potential during stationary phase in culture but not in the tick vector
Source: J Bacteriol. 2025 Feb 14;207(3):e00457-24. doi: 10.1128/jb.00457-24 (PMC11925233; doi:10.1128/jb.00457-24)
Supplement: Supplemental materials — Supplemental file information, Fig. S1 to S8, and supplemental references. [file jb.00457-24-s0003.pdf]

## SUPPLEMENTAL MATERIALS

# *Borrelia burgdorferi* loses essential genetic elements and cell proliferative potential during stationary phase in culture but not in the tick vector.

Jessica Zhang[1,2,†], Constantin N. Takacs[2,3,†,^], Joshua W. McCausland[2,3], Elizabeth A. Mueller[2,3], Jeline Buron[2,3], Yashna Thappeta[1,2], Jenny Wachter[4*], Patricia A. Rosa[4], Christine Jacobs-Wagner[1,2,3,5,#]

[1]Department of Biology, Stanford University, Stanford, CA 94305, USA

[2]Sarafan ChEM-H Institute, Stanford University, Stanford, CA 94305, USA

[3]Howard Hughes Medical Institute, Stanford University, Stanford, CA 94305, USA

[4]National Institutes of Health, Laboratory of Bacteriology, Rocky Mountain Laboratories, Division of Intramural Research, National Institute of Allergy and Infectious Diseases, Hamilton, MT 59840, USA

[5]Department of Microbiology and Immunology, Stanford University School of Medicine, Stanford, CA 94305, USA

† These authors contributed equally to this work.

*Present address: Vaccine and Infectious Disease Organization, University of Saskatchewan, Saskatoon, Canada

^Present address: Department of Biology, College of Science, Northeastern University, Boston, MA, USA

#Corresponding author: Jacobs-wagner@stanford.edu

**This PDF file includes:**

Supplementary file information

Supplementary figures S1 to S8

Supplementary references

## SUPPLEMENTARY FILE INFORMATION

**Supplemental File 1. Imaging Information.**
Reporting of specific exposure times and camera information for microscopy experiments, as well as specific n-values and intensityRatioThreshold parameters used during subsequent image analysis, are provided in this Excel file.

**Supplemental File 2. Source Data.**
Reporting of source data used to generate figures are provided in this Excel file.

Supplemental Files 1 and 2, along with all raw images (.nd2 or .raw16.tif file format) acquired and analyzed for this study are available on the public Biostudies repository (Accession S-BIAD1428 https://www.ebi.ac.uk/biostudies/bioimages/studies/S-BIAD1428).

## SUPPLEMENTARY FIGURES

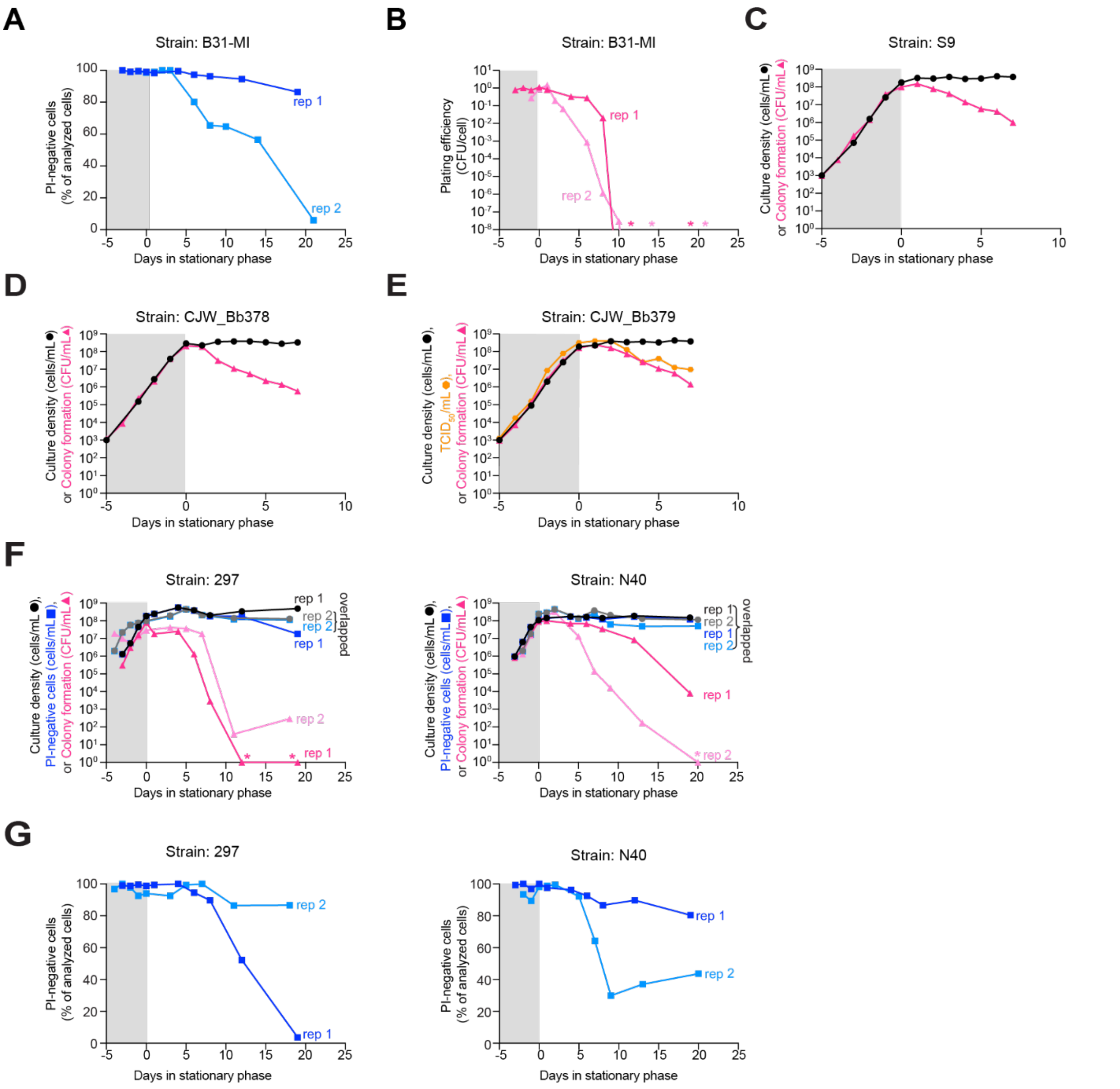


**Figure S1. Stationary phase phenotypes characterized in various strains.** Gray and white backgrounds indicate exponential and stationary phases, respectively. **A.** Plot showing the percentage of cells (n = 72 to 417 for each strain and time points; see Supplemental File 1 for specific n values) from cultures used in Fig. 1A that remained negative for propidium iodide (PI) staining shown on a linear scale; these values were used to calculate the number of PI-negative cells. Results from two independent cultures (biological replicates rep 1 and 2, represented by dark and light blue squares) of strain B31-MI are shown. **B.** Plot showing the plating efficiency for B31-MI. Plating efficiency was calculated by dividing the CFU/mL of a culture by the number of cells/mL measured at each time point (shown in Fig. 1A). Asterisks indicate that the plating efficiency could not be plotted on a log scale, as the value was zero. Results from two

independent cultures (biological replicates rep 1 and 2, represented dark and light pink triangles) of strain B31-MI are shown. **C.** Plot showing culture densities (cells/mL, black circles) in comparison to colony-forming ability (CFU/mL, pink triangles) for a single culture of the clonal B31-derived strain S9. **D.** Same as in (C) for clonal B31-derived strain CJW_Bb378. **E.** Same as in (C) but for clonal B31-derived strain CJW_Bb379. Here, the density of viable cells in the culture was also determined in liquid culture using a microtiter plate-based limiting dilution assay. This density of viable cells is expressed as tissue culture infectious dose 50 ($TCID_{50}$) per mL (shown as orange hexagons). **F.** Same as in Fig. 1A but for two independent cultures (biological replicates, rep 1 and 2) of strains 297 (left) and N40 (right). Black and gray circles represent culture density, dark and light blue squares represent PI-negative cells, and dark and light pink triangles represent CFU/mL for rep 1 and rep 2, respectively. Asterisks indicate that no colonies were detected. For PI-negative cell determination, 45 to 350 cells were analyzed for each strain and time point (see Supplemental File 1 for specific n values). **G.** Plots showing the percentage of PI-negative cells in the same cultures as used in (F) shown on a linear scale. For PI-negative cell determination, 45 to 350 cells were analyzed for each strain and time point (see Supplemental File 1 for specific n values).

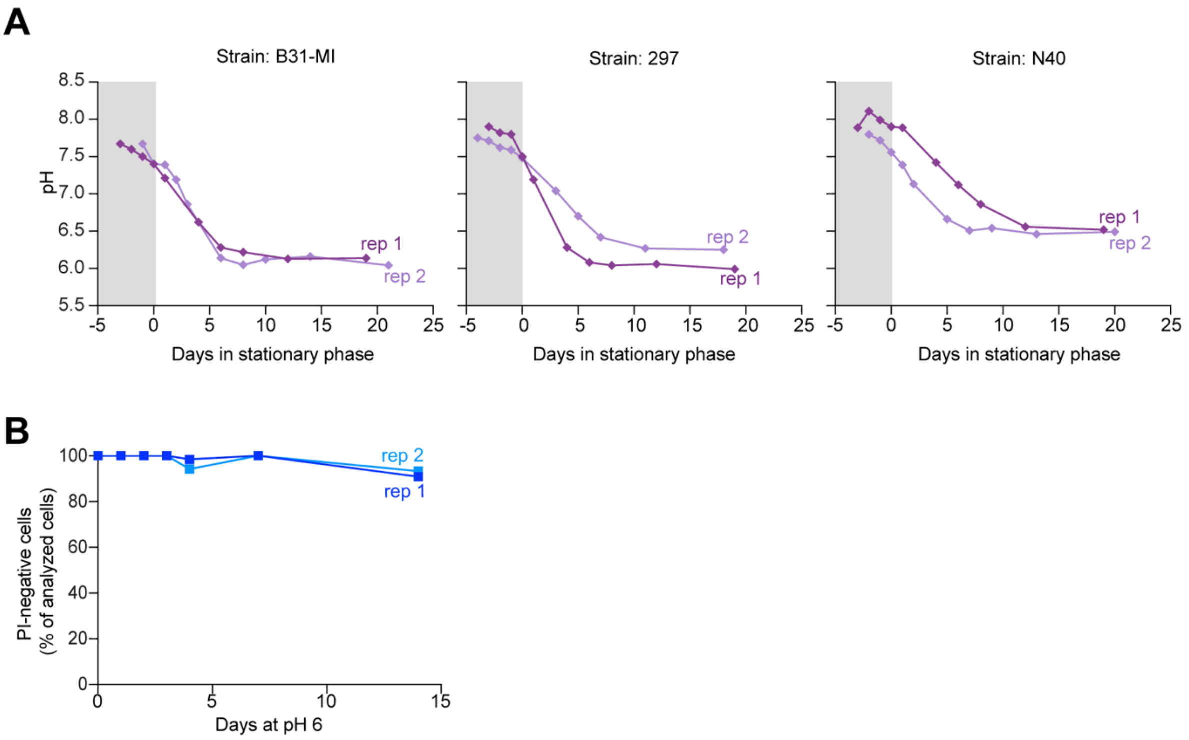


**Figure S2. Medium acidification in cultures and its effect on membrane permeability.**
**A.** Plots showing the changes in pH that occurred during cultivation of strains B31-MI, N40, and 297 in BSK-II medium. These pH measurements were done using the same cultures as those for Figs. 1 and S1F-G. Gray and white backgrounds indicate exponential and stationary phases, respectively. Shown are results from two independent cultures (biological replicates, rep 1 and 2) of each indicated strain. **B.** Plot showing the percentage of K2 cells that remained negative for propidium iodide (PI) uptake after culture at pH 6.0. There are the same results as in Fig. 2B, except on a linear scale.

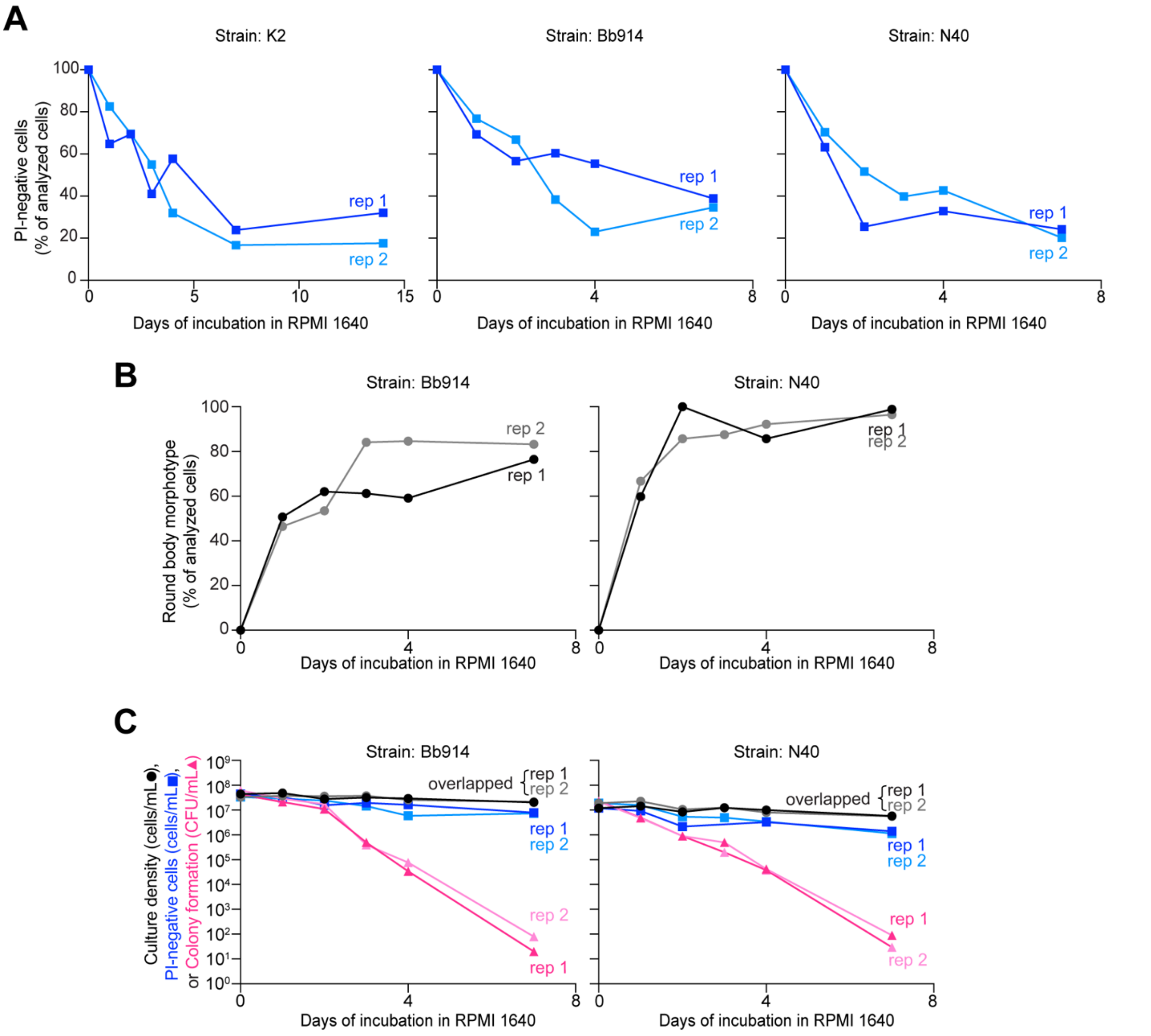


**Figure S3. Effects of starvation in RPMI 1640 on various *B. burgdorferi* strains.** Results from two independent cultures (biological replicates, rep 1 and 2) of each respective strain are shown. See Supplemental File 1 for specific n values for each time point and strain. **A.** Plots showing the percentage of cells from cultures of strain K2 (as used in Fig. 3), strain Bb914 (a derivative of strain 297), and non-clonal strain N40 that remained negative for propidium iodide (PI) uptake shown on a linear scale. For PI-negative cell determination, 48 to 247 cells were analyzed for each strain and time point. **B.** Same as Fig. 3C for cultures of strains Bb914 and N40. For round-body determinations, 70 to 311 cells were analyzed for each strain and time point. **C.** Same as Fig. 3D for cultures of Bb914 and N40. For PI-negative cell determination, 70 to 311 cells were analyzed for each strain and time point.

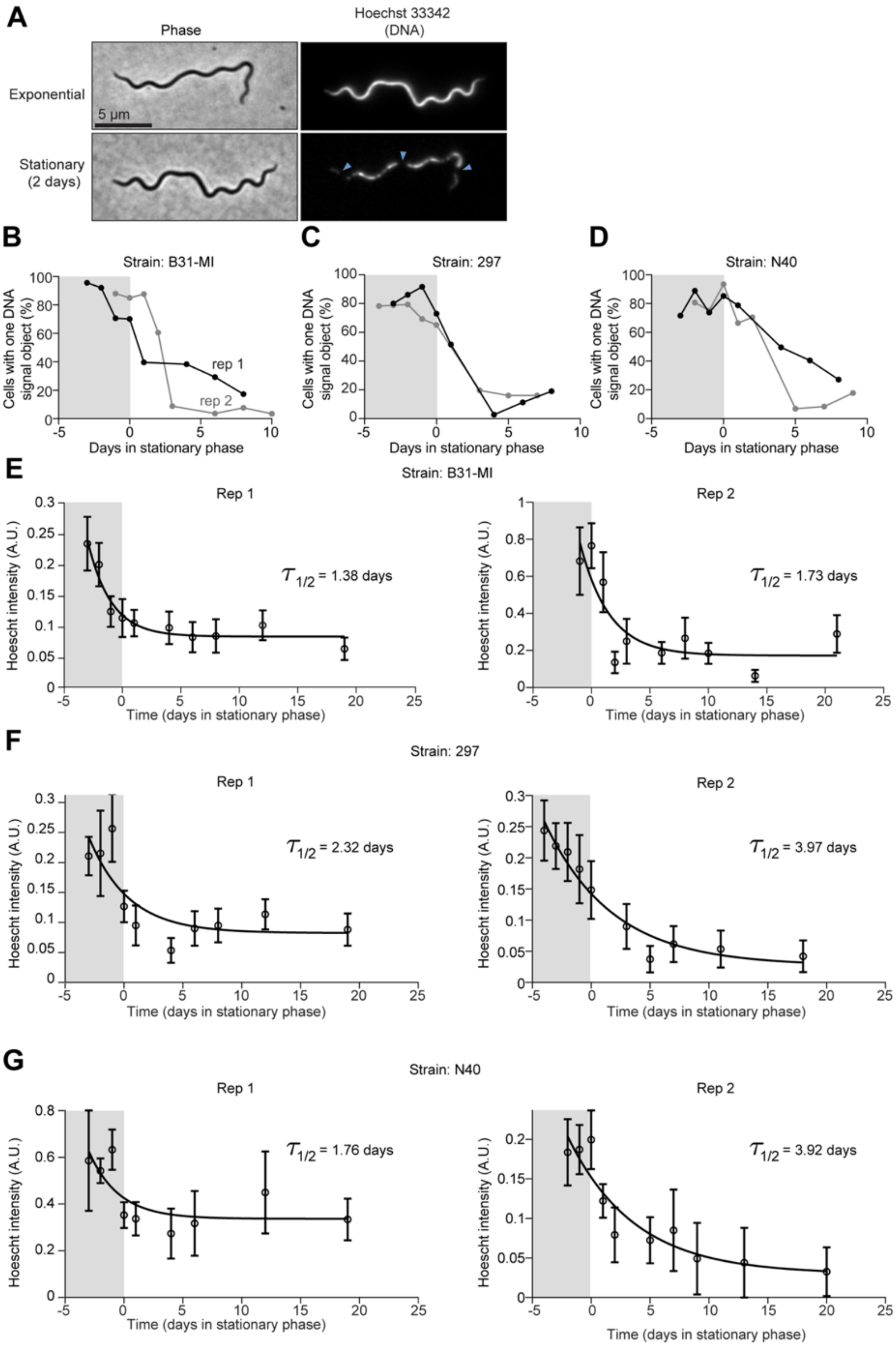


**Figure S4. Changes to DNA staining patterns in stationary phase cells.**
For panels B-G, gray and white backgrounds indicate exponential and stationary phases, respectively. Results from two independent cultures (biological replicates, rep 1 and 2) of each respective strain are shown. See Supplemental File 1 for specific n

values for each time point and strain. **A.** Representative images of a cell from an exponential or stationary phase culture of B31-MI where DNA was visualized by staining with Hoechst 33342. Light blue arrowheads indicate gaps depleted of DNA signal in the stationary phase cell. **B.** Plot showing the percentage of the cell populations with one continuous DNA signal for the B31-MI cultures used in Fig. 1. For DNA object detection analysis, 72 to 418 cells were analyzed for each time point. **C.** Same as in (B) except for cultures of strain 297, which were the same cultures as those used for Fig. S1F. For DNA object detection analysis, 66 to 332 cells were analyzed for each time point. **D.** Same as in (B) except for cultures of strain N40, which were the same cultures as those used for Fig. S1F. For DNA object detection analysis, 45 to 350 cells were analyzed for each time point. **E.** Plot showing the decay of the Hoechst signal intensity in BM31-MI cells as a function of culture age. Mean whole cell intensity of either biological replicate for each time point was fit to an exponential decay to illustrate the sharp decrease in signal. The same B31-MI cultures and cell outlines were used as in Fig. 1. A.U. indicates arbitrary units. **F.** Same as in (E) except for cultures of strain 297. **G.** Same as in (E) except for cultures of strain N40.

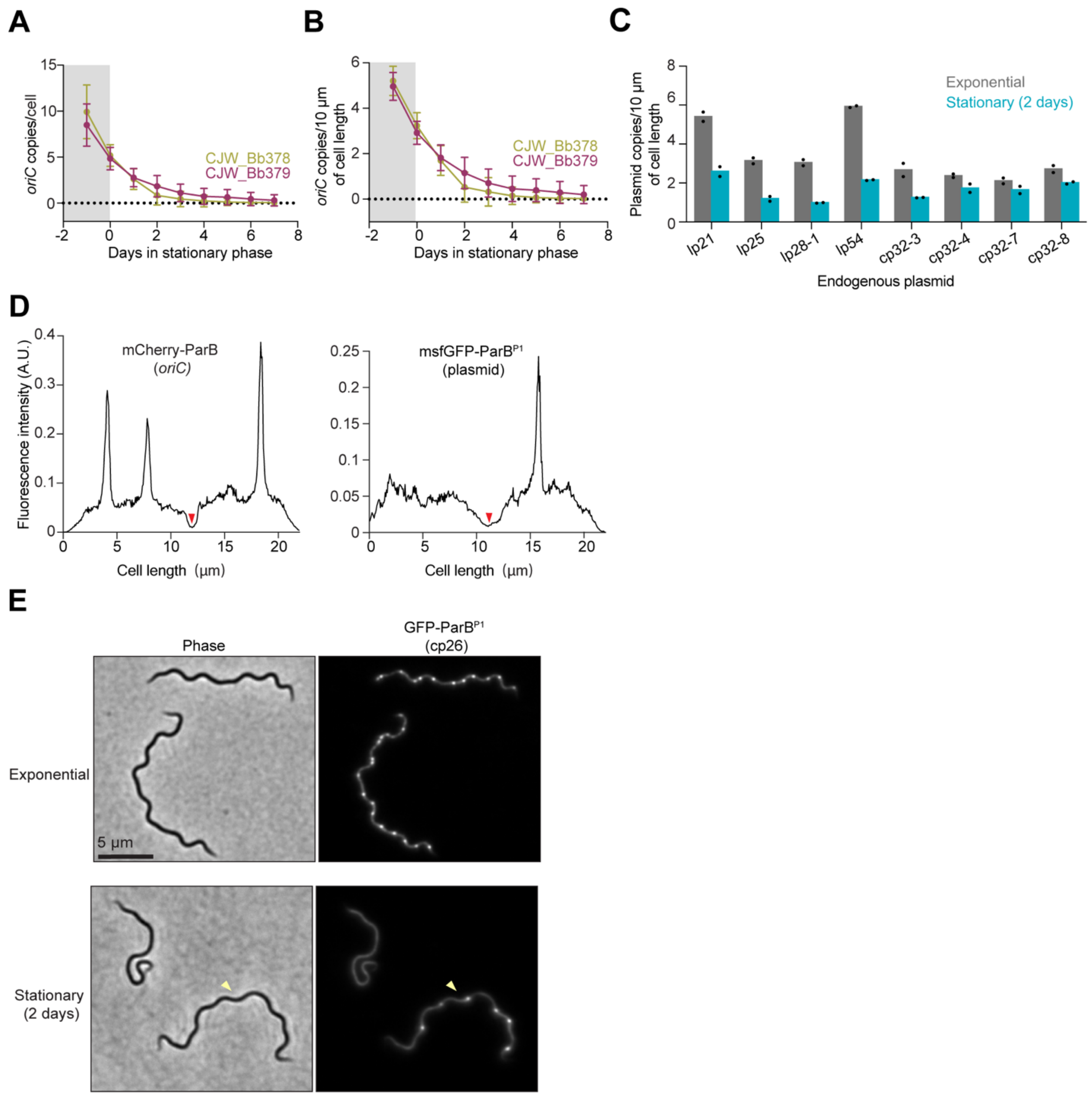


**Figure S5. Decrease in *oriC* and plasmid copy density during stationary phase.** **A.** Plot showing changes in *oriC* copies per cell in cultures of strains CJW_Bb378 and CJW_Bb379 (both clonal B31 derivatives) in BSK-II medium. The numbers of *oriC* copies were determined from fluorescence microscopy images by counting the fluorescent foci of mCherry-ParB (CJW_Bb379) or ParZ-msfGFP (CJW_Bb378) that indicate the subcellular location of the endogenous *parAZBS* region adjacent to *oriC* (1). One culture for each strain was analyzed at the indicated time points. Shown are means ± standard deviations across cells. For each strain and time point, 60 to 387 cells were analyzed (see Supplemental File 1 for specific n values). Gray and white backgrounds indicate exponential and stationary phases, respectively. **B.** Same as in (A) except that the mean *oriC* densities (expressed as *oriC* copies per 10 µm of cell length) are plotted. **C.** Plot showing plasmid densities (expressed as plasmid copy number per 10 µm of cell

length) in exponential phase (gray bars) and after two days in stationary phase (teal bars) using the same cultures as in Fig. 4C. Each black dot represents an independent biological replicate. Only cells (n = 22-167) with at least one clear *oriC* focus were considered in this analysis. The strain identities and the number of cells analyzed for each data point are detailed in Supplemental File 1. **D.** Plot showing the intensity profiles for mCherry-ParB and GFP-ParB$^{P1}$ signals along the cell length for the CJW_Bb489 cell shown in Fig. 4E. The division site, reflected by the dip in fluorescence signal, is indicated by red arrowheads. A.U. stands for arbitrary units. **E.** Representative phase contrast and fluorescence images of cells of strain CJW_Bb203 in which cp26 is labeled with msfGFP-ParB$^{P1}$. Cells were from a population in exponential phase or in stationary phase for two days. Yellow arrowheads point to a stationary phase cell with clear fluorescent cp26 foci.

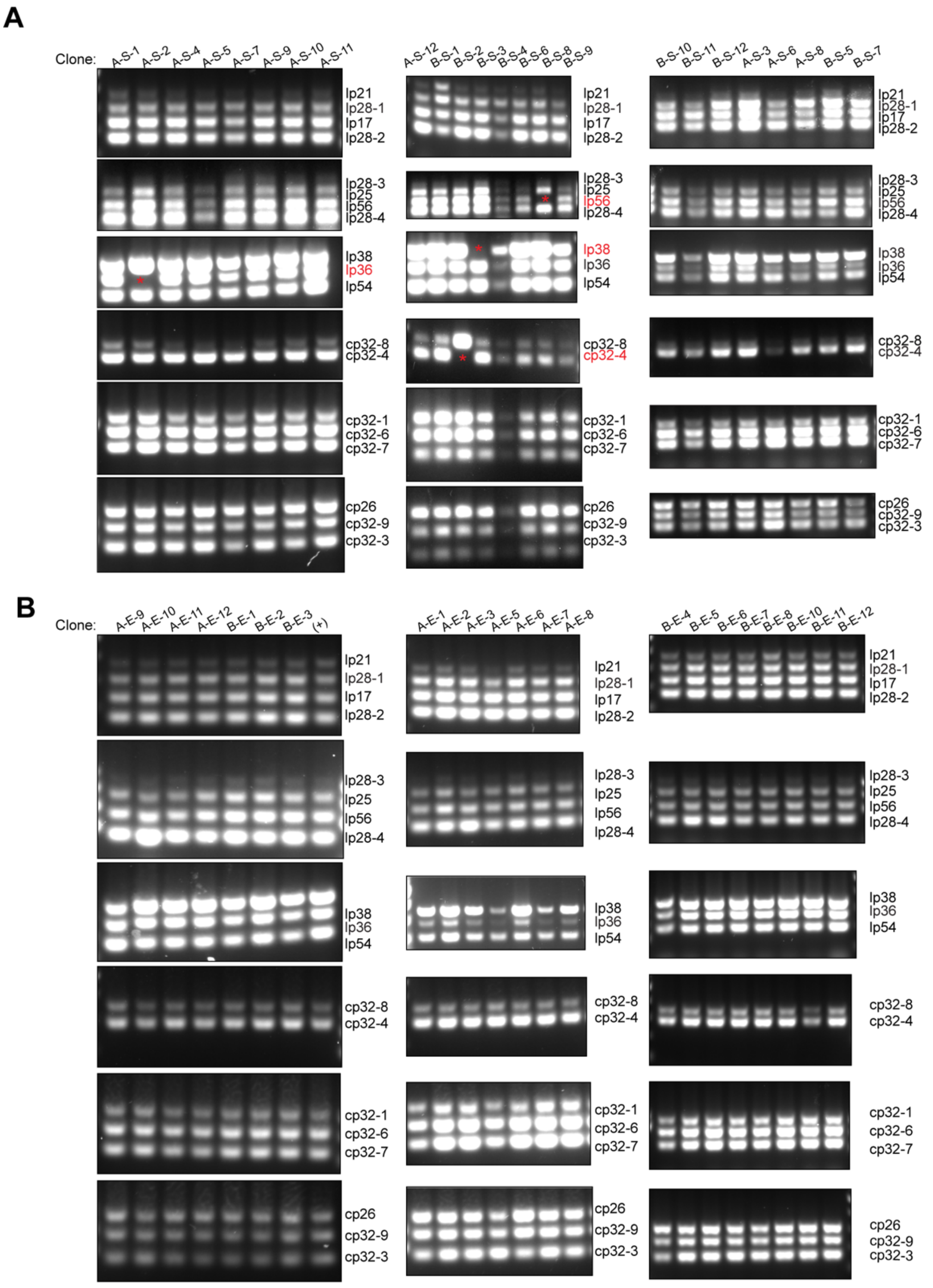


**Figure S6. Assessment of the plasmid content in *B. burgdorferi* clones isolated after growth in laboratory cultures.** Multiplex PCR was done using plasmid-specific primer pairs previously validated for use on strain B31 (2) and grouped in six sets, as shown in the images. The PCR products were separated by electrophoresis and visualized by SYBR Safe staining and automated detection using a Bio-Rad ChemiDoc Imaging System gel imager. The intensity of the resulting images was scaled to allow for visual detection of the weakly positive bands. As a result of acquisition and scaling, some of the more intense bands are saturated. All multiplex PCR results for all clones

analyzed are summarized in Table 1. **A**. Gel images for PCR products obtained by multiplex PCR profiling of all clones tested from 10-day-old stationary phase culture of CJW_Bb523. Lost plasmids (written in red) are indicated with a red asterisk on the gel image. Each clone tested is given a unique identifier: first A or B, corresponding to biological replicate 1 or 2, then S for stationary phase, followed by the identification number of the screened colony (see Table 1 for details). **B.** Same as in (A) except that from cultures in exponential phase. Each clone tested is given a unique identifier as described in (A) except that E is for exponential phase. The (+) indicates a positive control sample, which corresponds to strain CJW_Bb523 isolated in exponential phase.

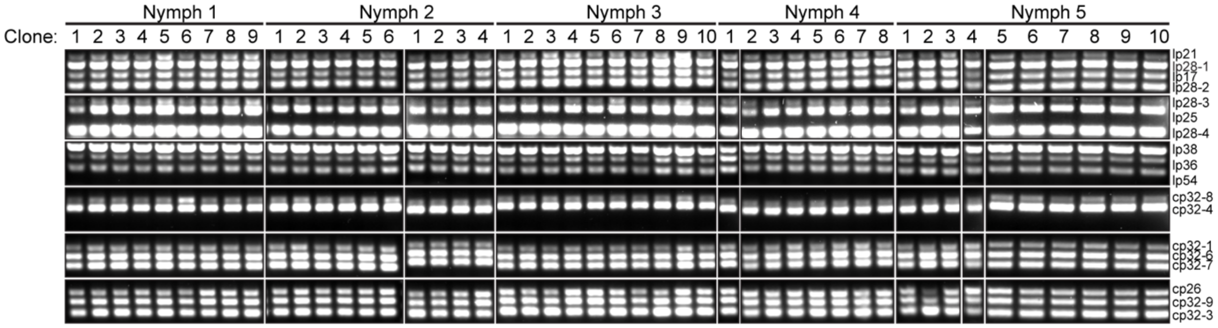


**Figure S7. Assessment of the plasmid content in *B. burgdorferi* clones isolated from unfed ticks.** Same as in Fig. S6 except that the clones were isolated from the unfed ticks used for Fig. 5. Shown are gel images of PCR products from clones obtained by plating crushed nymphs colonized with strain CJW_Bb474 and maintained unfed at room temperature for 14 months after molt. CJW_Bb474 is a clonal B31-MI derivative that lacks lp5, cp9, and lp56. Isolated and tested clones are grouped by nymph.

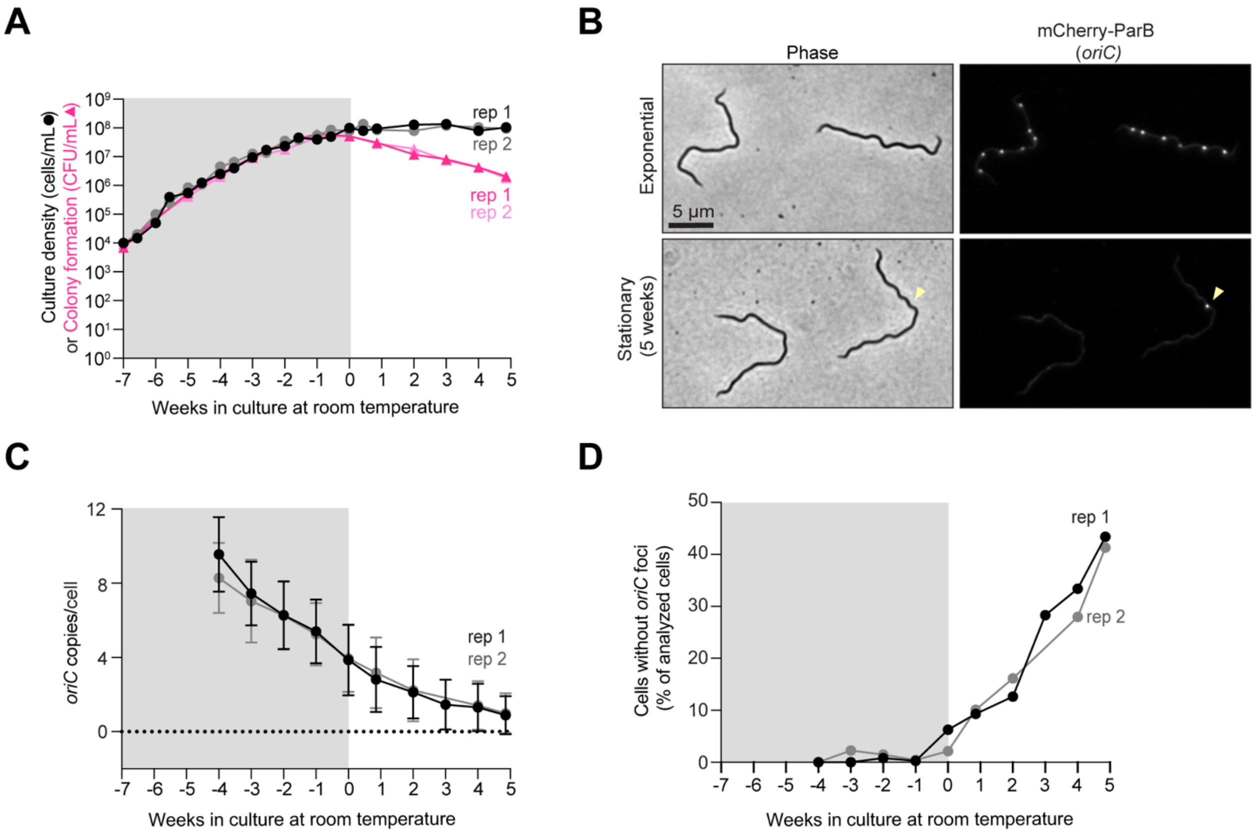


**Figure S8. Determination of cell proliferative potential and *oriC* copy number per cell for cultures grown at room temperature.**

**A.** Plot showing culture densities (cells/mL, black and gray circles) and colony forming ability (CFU/mL, dark and light pink triangles) for two independent cultures (rep 1 and rep 2) of CJW_Bb379 grown in BSK-II at room temperature (~21°C). Due to the slower growth rate associated with the lower temperature, the x-axis is standardized relative to weeks in stationary phase. Gray and white backgrounds indicate exponential and stationary phases, respectively. **B.** Representative phase contrast and fluorescence images of CJW_Bb379 cells in which the *oriC* region is labeled with mCherrry-ParB. Cells were imaged in exponential phase or after five weeks in stationary phase. Yellow arrowheads point to the stationary phase cell with a clear fluorescently labeled *oriC* focus to contrast with the neighboring cell lacking a fluorescent spot. **C.** Plot showing changes in *oriC* copies per cell over time for the two cultures of CJW_Bb379 shown in (A) and grown in BSK-II at room temperature (~21°C). For *oriC* copy number quantification, 7 to 811 cells were analyzed for each strain and time point (see Supplemental File 1 for specific n values for each time point). Gray and white backgrounds indicate exponential and stationary phases, respectively. **D.** Plot showing the percentage of the cell populations without clear fluorescent *oriC* foci, using the same cultures as in (A-C). Gray and white backgrounds indicate exponential and stationary phases, respectively. For each time point, 7 to 811 cells were analyzed (see Supplemental File 1 for specific n values).

## SUPPLEMENTARY REFERENCES

1. Takacs CN, Wachter J, Xiang Y, Ren Z, Karaboja X, Scott M, Stoner MR, Irnov I, Jannetty N, Rosa PA, Wang X, Jacobs-Wagner C. 2022. Polyploidy, regular patterning of genome copies, and unusual control of DNA partitioning in the Lyme disease spirochete. Nat Commun 13:7173.

2. Bunikis I, Kutschan-Bunikis S, Bonde M, Bergström S. 2011. Multiplex PCR as a tool for validating plasmid content of Borrelia burgdorferi. J Microbiol Methods 86:243–247.
